# The level of origin of renal arteries in horseshoe kidney vs. in separated kidneys: CT-based study

**DOI:** 10.1007/s00276-018-2071-8

**Published:** 2018-07-24

**Authors:** Marcin Majos, Michał Polguj, Zofia Szemraj-Rogucka, Agata Arazińska, Ludomir Stefańczyk

**Affiliations:** 1 Department of Radiology, Barlicki University Hospital, Medical University of Łódź, Kopcińskiego Str. 22, 90-153 Lodz, Poland; 2 Department of Angiology, Medical University of Łódź, Narutowicza Str. 60, 90-136 Lodz, Poland; 3 Department of Radiological and Isotopic Diagnosis and Therapy, Medical University of Łódź, ul. Pomorska 251, 92-213 Lodz, Poland

**Keywords:** Renal artery, Multiple, Horseshoe kidney, Computed tomography angiography

## Abstract

**Purpose:**

Horseshoe kidney is a rare congenital anomaly with potential clinical implications. The aim of this study was to determine the number of renal arteries and veins and the level at which the arteries branched off their parental vessels in individuals with horseshoe kidney (HSK) and in persons with separated kidneys (SK).

**Materials and methods:**

The analysis included computed tomography angiography studies of 331 patients (83 HSK and 248 SK). The number of renal vessels and diameters of renal arteries were determined, along with the level at which they branched in relation to other ramifications (four groups of origin were proposed) and their entrance of the vessels to the kidney.

**Results:**

Number of renal arteries in HSK group was 4.57 ± 1.39 per patient and 2.4 ± 0.43 in SK group (*p* < 0.0001). The distribution of branching level of renal arteries in HSK group was: I group ~ 57%, II group ~ 27%, III group ~ 15% and IV group < 1%, whereas in SK group the distribution was respectively: I group ~ 99%, II group < 1%, III and IV group − 0% (*p* = 0.0001). In HSK group, diameter of renal arteries branching above the IMA was 4.61 ± 1.58 mm, as compared with 3.96 ± 1.34 mm for the arteries branching below (*p* = 0.0004). Number of veins was 566 in SK group (87.70% of kidneys were supplied by single vein) and 323 in HSK group (9.64% kidneys were supplied by two veins) (*p* < 0.0001).

**Conclusion:**

In HSK group, renal arteries significantly more often branch off their parental vessels below the origin of IMA and such vessels are usually smaller.

## Introduction

Horseshoe kidney is a rare congenital anomaly resulting from abnormal differentiation of the mesonephros. The number of horseshoe kidneys differs according to various authors, and are supposed to be found in approximately 1 per 400 to 1 per 800 newborns [6, 17, 35, 37]. This implies that surgeons may often deal with this anomaly during the course of their everyday practice.

Renal vasculature is in centre of scientific interest for long time [27] and is well described [1, 3, 13, 31]; however, previous analysis should be revised because along with post-mortem examinations [19] new techniques were adapted [10, 30]. Despite some attempts in this matter, vascular network of horseshoe kidney has still not been fully described, primarily due to its considerable variability [7]. A systematic analysis of horseshoe kidney vasculature would with no doubt facilitate many abdominal and pelvic surgeries, in particular abdominal aortic aneurysm treatment [15], iliac artery angioplasty or bypass surgery [12, 14], renal artery angioplasty and kidney transplantation [5, 24]. A key morphological feature which with no doubt needs to be considered during such analysis is the level at which renal arteries branch off their parental vessels.

The aim of this study was to determine the number of renal arteries and veins as well the level at which arteries branched off their parental vessels, where arteries entered the kidney and where the renal veins originated from in individuals with horseshoe kidney and in persons with separated kidneys. The origins of renal arteries were classified using an original 4-grade system developed solely for the purpose of this study. Moreover, we verified if the renal arteries for horseshoe kidneys differed in terms of their diameters from the vessels supplying separated kidneys.

## Materials and methods

### Material

The study was based on a retrospective analysis of images stored at PACS archiving system at the Department of Radiology, University Clinical Hospital No. 1 in Łódź. The analysis included images from all consecutive patients in whom computed tomography (CT) angiography of the abdominal aorta demonstrated presence of horseshoe kidney (between January 2006 and January 2017) or separated kidneys (between March 2016 and January 2017). The only inclusion criterion of the study was the presence of a single horseshoe kidney (horseshoe kidney group) or two normally developed kidneys (control group). The list of exclusion criteria comprised a history of surgical procedures involving complete or partial resection of the kidney, kidney transplantation or renal artery angioplasty, poor quality or inadequate CT angiographic images (lack of kidney components on the image, insufficient contrast enhancement, motor artifacts or other artifacts hindering comprehensive evaluation of renal arteries, e.g. metallic hardware in the spine or barium contrast in the intestines).

The horseshoe kidney group included 83 persons, among them 32 women and 51 men. Mean age of the study group was 65.6 years (SD = 16.23), with range between 15 and 96 years, median amounting to 65.5 years, and lower and upper quartile equal 57 and 79 years, respectively (Table 1). The control group was comprised of 248 subjects (122 women and 126 men) with two separated kidneys. Mean age of the controls was 66.4 years (SD = 15.01), with range between 24 and 94 years, median of 68 years, and lower and upper quartile equal 58 and 78 years, respectively (Table 1).

**Table 1:** Demographic data of patients with horseshoe kidney (HSK) and with sepatated kidneys (SK)

	*N*	Mean age (years)	Median (years)	SD (years)	Min (years)	Max (years)	I quartile (years)	III quartile (years)
HSK	83	65.6	65.5	16.23	15	96	57	79
SK	248	66.4	68.0	15.01	24	94	58	78

The protocol of the study was approved by the Local Bioethics Committee at the Medical University of Łódź, Poland (decision no. RNN/132/17/KE of 11 April 2017).

### Method

**Fig. 1:**
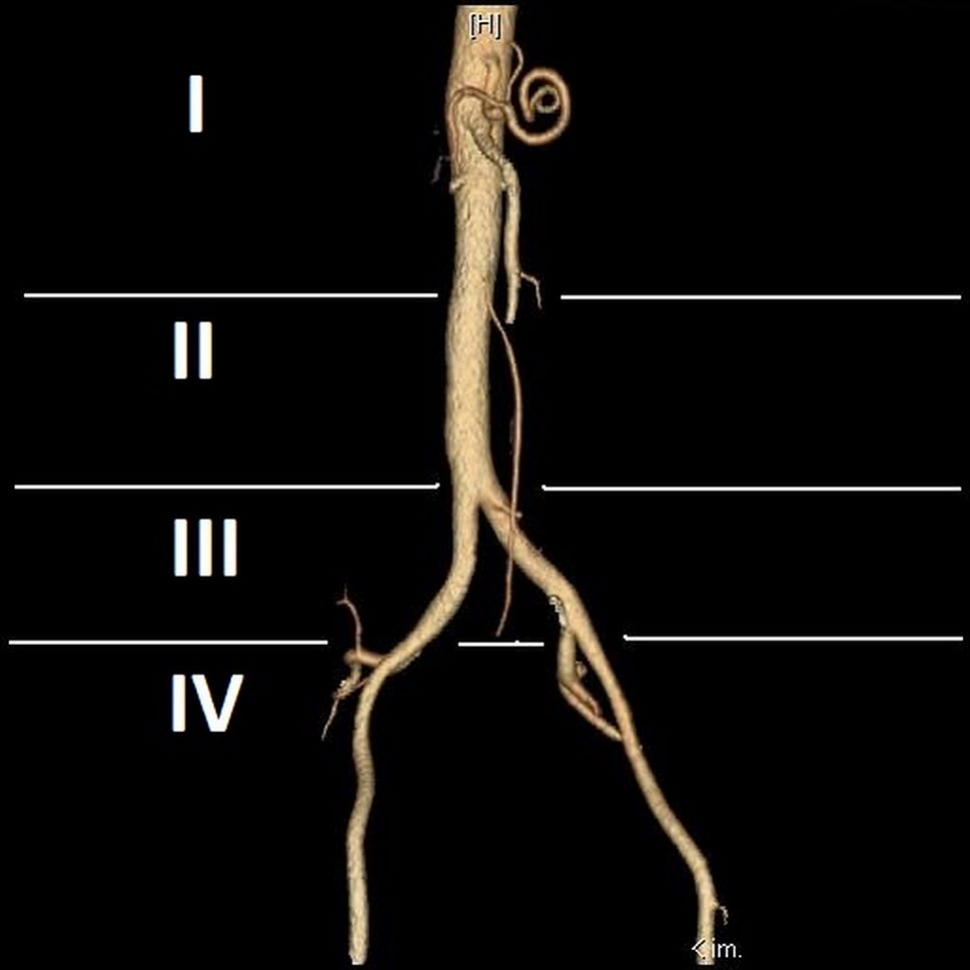
Diagram of division of renal arteries to specific groups on a base of level of their origin

CT angiography was performed with GE Light Speed 64 VCT scanner (GE Healthcare, Milwaukee, WI, USA; kV 120, mA 10, mAs dynamic), with 0.625-mm layer width and 0.6-mm pitch, after intravenous administration of 80–100 ml of Ultravist 370 contrast agent (BAYER Schering Pharma AG, Germany) with an automatic syringe at a flow rate of 4.5–4.2 ml/s. Transverse and frontal CT angiographic images were evaluated at a doctor’s console with an aid of AW 4.0 GE software. The number of renal vessels related with each kidney was determined; in patients with horseshoe kidney, this structure was classified as a right- or left-sided using the smallest sagittal cross-section of the isthmus as a topographic landmark. Renal arteries supplying the isthmus, especially those that ramified before entering the renal parenchyma, were qualified as right- or left-sided if they branched off their parental vessels on the right or left side of the aortic axis, respectively. The level at which a renal artery branched off the aorta or iliac artery was described in relation to other ramifications of those parental vessels. Based on this criterion, the origin of each renal artery was classified into one out of four groups: I—above the inferior mesenteric artery, II—between the inferior mesenteric artery and the aortic bifurcation, III—between the aortic bifurcation and the bifurcation of the common iliac artery, and IV—below the bifurcation of the common iliac artery (Fig. 1). Diameter of each renal artery was measured 15 mm distally from its parental vessel. The anatomical variants of arterial entrances were determined as single renal artery (SA) passing through the hilum, accessory renal arteries (ASA) also passing through the hilum, parenchymal renal arteries (PA) which omit kidney hilum but reach parenchyma close to it and polar renal arteries (PRA) entering renal parenchyma in upper or lower 1/6 of a kidney. In HSK group we considered as SA variants arteries supplying one renal unit. The renal veins were divided to two anatomical groups single renal veins (SV) originating in the hilum and accessory renal veins (ASV). As in the arteries, in HSK group the veins originating from renal hilum, one from each renal unit, were considered as SV variant.

### Statistical analysis

Statistical characteristics of quantitative variables were presented as means, standard deviations (SD), medians, minimum and maximum values, lower and upper quartiles. Before the intergroup comparison of each quantitative variable, normality of its distribution was verified with Shapiro–Wilk test. Since the distributions of the study variables in the horseshoe kidney group were not normal, the significance of intergroup differences was verified with non-parametric Mann–Whitney *U* test.

## Results

**Fig. 2:**
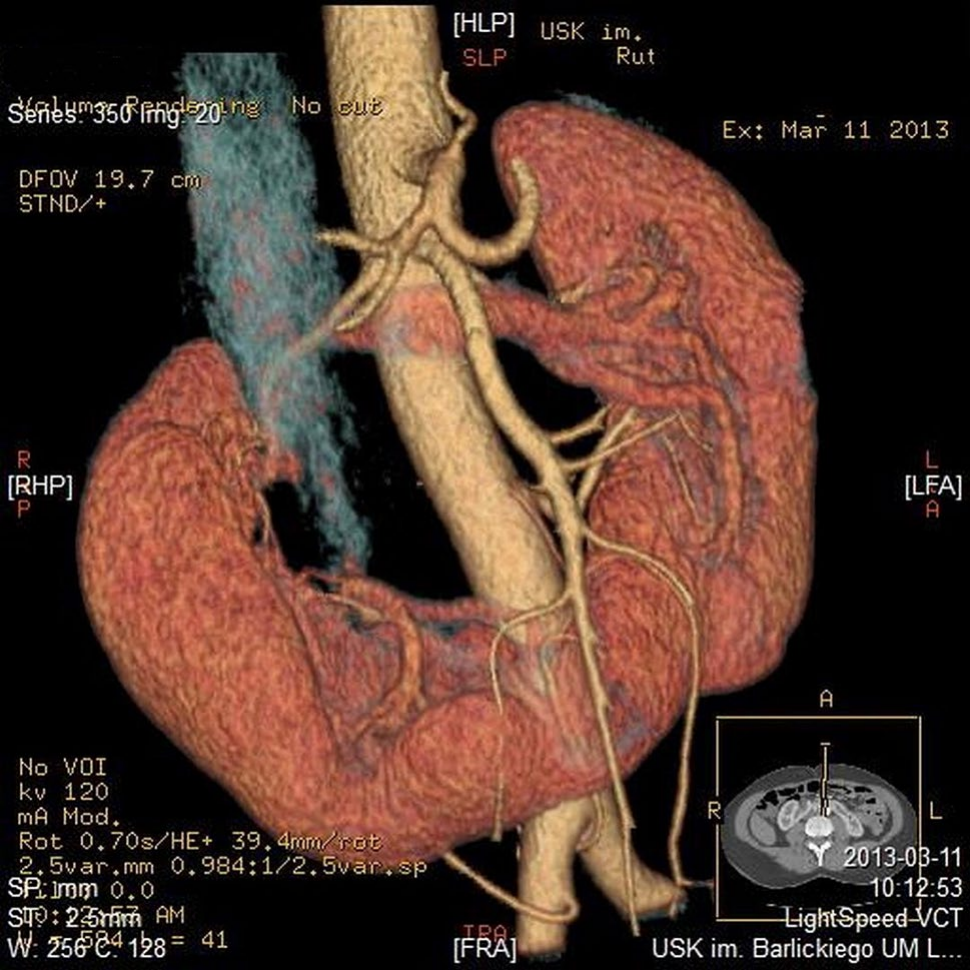
Patient with horseshoe kidney supplied by five renal arteries, that includes one originating below inferior mesenteric artery (group II) and one originating from right common iliac artery (group III)

**Fig. 3:**
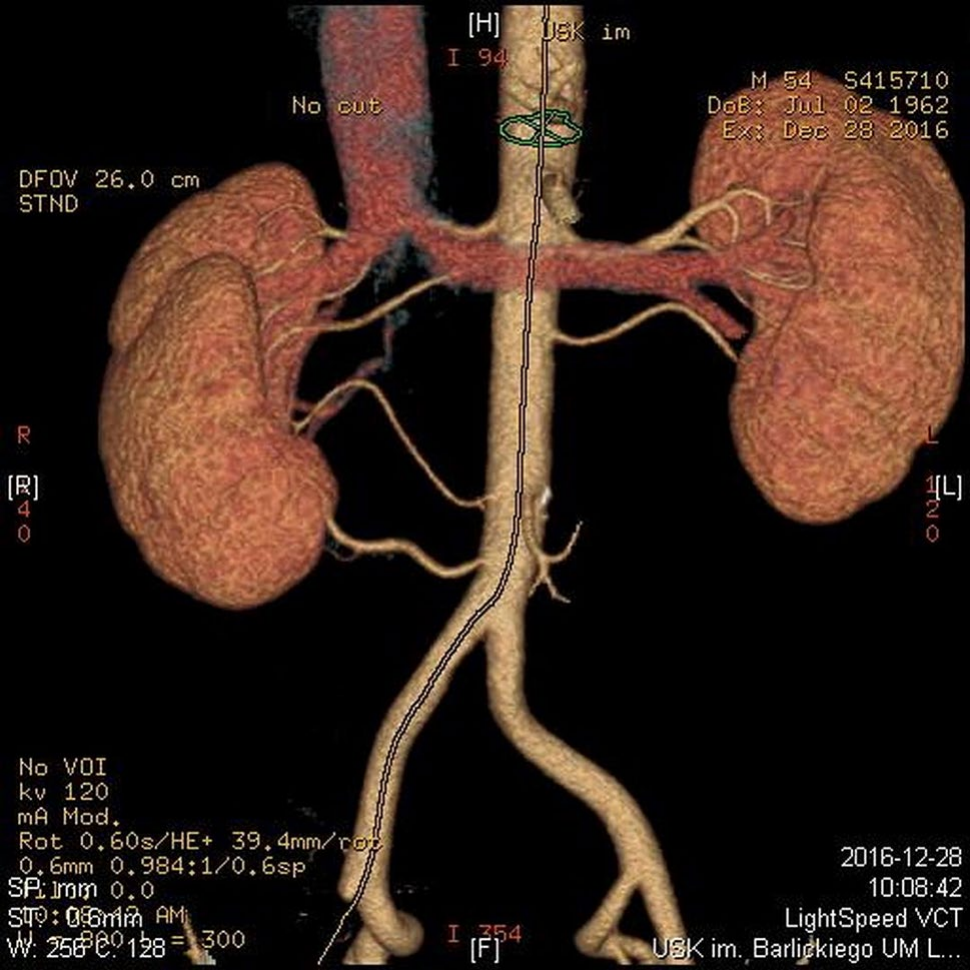
Patient with separated kidneys supplied by six renal arteries (four supplying right and two supplying left kidney). One of them originating inferiorly to inferior mesenteric artery (group II)

A total of 379 renal arteries were identified in the horseshoe kidney group, with 4.57 ± 1.39 arteries per subject on average. Of this number, 194 renal arteries were classified as right-sided as they supplied renal parenchyma located right of the midline or the reached renal isthmus after branching off on the right side of the aortic axis (Fig. 2). Other 185 renal arteries supplied horseshoe kidneys located left of the midline, or branched off on the left side of the aortic axis and run towards the renal isthmus (Fig. 2). The difference in the prevalence of right- and left-sided renal arteries supplying horseshoe kidneys was not statistically significant (*p* = 0.5827). In HSK group the number of kidneys supplied by SA variant was 3 (3.61%), 19 (22.89%) by ASA variant, 29 (34.93%) by PA and 32 kidneys (38.55%) with PRA variant. In comparison SK group we indentified 400 kidneys (80.64%) supplied by SA variant, 79 kidneys (15.93%) supplied by ASA, 11 kidneys (2.22%) by PA variant and only 6 kidneys (1.21%) with vessels representing PRA variant (Fig. 3).

Overall number of renal arteries identified in the control group amounted to 598, with a mean number of 2.4 ± 0.43 arteries per subject. This subset included 304 arteries for the right kidney and 294 arteries supplying the left kidney. Similar to the horseshoe kidney group, no statistically significant difference was found in the prevalence of right- and left-sided renal arteries in the controls (*p* = 0.3474).

The study groups differed significantly in terms of the number of renal arteries (*p* < 0.0001). While in the control group, mean number of renal arteries for the left kidney turned out to be significantly higher than the mean number of renal arteries for the right kidney (*p* = 0.0727), no statistically significant differences in the number of right- and left-sided renal arteries were found in the horseshoe kidney group (*p* = 0.677). The results are presented in Table 2.

**Table 2:** Number of renal arteries supplying horseshoe kidneys and separated kidneys depending on sides

	Number of renal arteries	
	Horseshoe kidneys (HSK)	Separated kidneys (SK)
Right side	194 (2.34)	304 (1.23)
Left side	185 (2.23)	294 (1.19)

Out of 379 renal arteries for horseshoe kidneys, 217 (57.26%) branched off their parental vessels above the origin of the inferior mesenteric artery (group I). A total of 103 renal arteries for horseshoe kidneys (27.18%) were classified to group II and 56 (14.78%) to group III. Only three renal arteries (less than 1%) had their origin below the bifurcation of the common iliac artery. The subset of 598 arteries supplying separated kidneys included 596 vessels (99.67%) that branched off the aorta above the origin of the inferior mesenteric artery (group I), and only two arteries representing topographic group II (less than 1%). None of the renal arteries for separated kidneys was classified to group III or IV. This difference turned out to be statistically significant (*p* = 0.0001). Detailed results are presented in Table 3.

**Table 3:** Number of renal arteries supplying horseshoe kidneys and separated kidneys depending on level of origin

Level of origin	Horseshoe kidney renal arteries HSK (%)	Separated kidney renal arteries SK (%)
I	217 (57.26)	596 (99.67)
II	103 (27.18)	2 (< 1)
III	56 (14.78)	0
IV	3 (< 1)	0

The analysis of ROC curve demonstrated that renal arteries for separated kidneys could be optimally distinguished from those supplying horseshoe kidneys on the basis of their relation to the origin of the inferior mesenteric artery. While up to 42.72% (*n* = 162) of renal arteries for horseshoe kidneys branched off the aorta or iliac artery below the origin of the inferior mesenteric artery, this topographic pattern was followed by less than 1% (*n* = 2) of the renal arteries supplying separated kidneys.

Mean diameter of renal arteries that branched off their parental vessels above the origin of the inferior mesenteric artery was 4.61 ± 1.58 mm, as compared with 3.96 ± 1.34 mm for the arteries that branched off below this topographic landmark; the difference was statistically significant (*p* = 0.0004). A total of 305 (80.47%) renal arteries had more than 3 mm in diameter. This subset included 184 renal arteries that branched off their parental vessel above the origin of the inferior mesenteric artery (84.79%) and 121 arteries that branched off below (74.69%); also this difference turned out to be statistically significant (*p* = 0.0004).

A total number of 323 veins were identified in HSK group with 3.89 ± 1.26 vein per subject. For comparison there were 566 veins localized in SK group with 1.13 ± 0.37 vein per subject on average. The analysis of anatomical variants of venal system revealed that in HSK group only 9 kidneys (10.84%) presented SV anatomical variant and 74 kidneys (89.16%) were characterized by more than two renal veins. On the other hand vast majority of kidneys in SK group (435 kidneys − 87.70%) were drained by only one vein when only in 61 kidneys (12.30%) presented ASV.

## Discussion

Separated kidneys have three stages of development during embryological period: pronephros, mesonephros and metanephros while they are ascending from pelvis to the typical localization [9, 15]. Horseshoe kidneys have similar fazes of development however they start to differ from separated kidneys from 4th week of gestation. According to the mechanical fusion theory, the extremities of horseshoe kidney merge during mesonephros stage when they are relatively close to each other in pelvis [29]. On the consequence their passage to typical, renal location is blocked by the presence of inferior mesenteric artery at a level of L3 [33]. What is more, existence of isthmus in horseshoe kidney not only terminates its ascension prematurely but also prevents its typical rotation [29].

Eventhough, the horseshoe is the most common type of renal developmental anomaly; although it does not usually constitute a primary cause of clinically relevant ailments, it may predispose to some complications if co-existing with other comorbidities. This refers not only to renal hypertension, urinary tract infections and nephrolithiasis, but also to other abdominal and pelvic diseases that require surgical intervention or endovascular treatment [2, 4, 8]. Presence of horseshoe kidney should be in particular considered during the treatment of aortic and iliac artery aneurysms, and during other procedures involving renal parenchyma [22, 36, 40]. A surgeon preparing for such procedures or even planning a thick needle biopsy, should be always aware of potential presence of extra renal arteries supplying the every kidney originating from various sources [28] as well as horseshoe kidney [26].

In our series, the number of renal arteries supplying horseshoe kidneys turned out to be nearly twice as high as the number of the arteries for separated kidneys (4.57 vs. 2.4 per patient). This observation is consistent with the findings reported by Ichikawa et al. [23] and Stroosma et al. [37]; some slight discrepancies between our observations and the results published by those authors may result from different ethnic backgrounds and sizes of the study groups.

Our study presented that characteristic for horseshoe vascular system is the vast widespread of accessory renal arteries and the high frequency of the renal arteries entering the kidney directly to parenchyma avoiding renal hilum. The other authors support our observation that accessory arteries, polar arteries and parenchyma arteries are not so common in separated kidneys [33, 34].

We demonstrated that the number of renal arteries that branched off the aorta below the origin of the inferior mesenteric artery in subjects with horseshoe kidney was considerably higher than in persons with separated kidneys. Alsco according to Glodny et al. [18], extra renal arteries frequently branch off the aorta below lumbar vertebra L3, i.e. below the typical origin of the inferior mesenteric artery [16]. Unfortunately, authors did not describe the topography of iliac arteries as precisely as the main ramifications of the aorta [11, 16], which precludes direct comparison of their findings with our results. Noticeably, a large proportion of extra renal arteries that branched off below the origin of the inferior mesenteric artery were derivatives of iliac vessels (*n* = 59). In three cases, extra renal artery branched off the aortic bifurcation, and in one it was a derivative of the inferior mesenteric artery; however, all those modifications should be considered as individual anomalies, rather than as established topographic variants.

We did not find a statistically significant relationship between the prevalence of extra renal arteries and the location of the horseshoe kidney in relation to the midline, which stays in opposition to the results published by Taghavi et al. [38]. Available data on the prevalence of right- and left-sided extra renal arteries in individuals with separated kidneys are inconclusive, and likewise in the case of horseshoe kidneys, those vessels are not always observed and their presence seems to be population-specific [20, 21].

This is quite important owing that the inferior mesenteric artery is either ligated during the course of open surgeries for abdominal aortic aneurysms or covered with a sent graft during the course of endovascular procedures. Also bypass treatment and angioplasty of the iliac vessels may result in obliteration of extra renal arteries that branch off at this level. It is still unclear whether it may impair the renal function and to what degree.

When the diameter of renal arteries was stratified according to their origin, the vessels that branched off above the inferior mesenteric artery turned out to be larger than those branching off below this topographic landmark.

We also determined the prevalence of renal arteries for horseshoe kidneys, which had more than 3 mm in diameter. Recent evidence suggests that the occlusion of an extra renal artery with diameter up to 3 mm does not interfere considerably with renal perfusion [25, 32, 39]. Consequently, implantation of a vascular stent to such vessel should not pose a risk of complications. Therefore, identification of renal arteries the diameters of which exceed that critical value seems to be important from a practical perspective. However, whether occlusion of more than one extra renal artery, even with a smaller diameter, may influence the secretory function of the kidney, is still a matter of discussion.

The proportion of renal arteries for horseshoe kidneys, which had more than 3 mm in diameter was higher than in a previous study conducted by Ichikawa et al. [23]: 80.47 vs. 69.2%, respectively. This discrepancy might result either from different demographic characteristics or larger size of the group examined by Ichikawa et al. [23] (*n* = 39).

We also analyzed the prevalence of renal arteries with more than 3 mm in diameter depending on the level at which they branched off their parental vessels. Although the proportion of larger renal arteries that branched off above the inferior mesenteric artery was substantially higher (84.79%), also the percentage of large vessels having their origin below this topographic landmark was relatively high (74.69%).

The venosus system in HSK group is much more complex than this described for SK group as ASV anatomical variant is more frequently found draining horseshoe kidneys (89.16% in HSK group vs. 12.30% in SK group). The observations of Satypal K. S. [35] support our anatomical findings in group of separated kidneys showing uniqueness of horseshoe kidney venosus system.

## Conclusions

In persons with horseshoe kidneys, renal arteries significantly more often branch off their parental vessels below the origin of the inferior mesenteric artery. Such vessels are usually smaller than those branching off above this topographic landmark, but still many of them may have diameters greater than a clinically important 3-mm threshold.
